# The FLASSH study: protocol for a randomised controlled trial evaluating falls prevention after stroke and two sub-studies

**DOI:** 10.1186/1471-2377-9-14

**Published:** 2009-03-31

**Authors:** Frances A Batchelor, Keith D Hill, Shylie F Mackintosh, Catherine M Said, Craig H Whitehead

**Affiliations:** 1 National Ageing Research Institute, PO Box 2127, The Royal Melbourne Hospital, Parkville, Victoria 3050, Australia; 2 School of Physiotherapy, University of Melbourne, Parkville, Victoria 3052, Australia; 3 Musculoskeletal Research Centre, Faculty of Health Sciences, Latrobe University and Northern Health, Bundoora, Victoria 3083, Australia; 4 School of Health Sciences, University of South Australia, Adelaide, South Australia 5000, Australia; 5 Rehabilitation Sciences Research Centre, School of Physiotherapy, University of Melbourne, c/o Royal Talbot Rehabilitation Centre, Kew Victoria 3101, Australia; 6 Heidelberg Repatriation Hospital, Austin Health, Heidelberg, Victoria 3081, Australia; 7 Division of Rehabilitation, Aged Care and Allied Health, Repatriation General Hospital, Daw Park, South Australia 5043, Australia

## Abstract

**Background:**

Falls are common in stroke survivors returning home after rehabilitation, however there is currently a lack of evidence about preventing falls in this population. This paper describes the study protocol for the FLASSH (FaLls prevention After Stroke Survivors return Home) project.

**Methods and design:**

This randomised controlled trial aims to evaluate the effectiveness of a multi-factorial falls prevention program for stroke survivors who are at high risk of falling when they return home after rehabilitation. Intervention will consist of a home exercise program as well as individualised falls prevention and injury minimisation strategies based on identified risk factors for falls. Additionally, two sub-studies will be implemented in order to explore other key areas related to falls in this population. The first of these is a longitudinal study evaluating the relationship between fear of falling, falls and function over twelve months, and the second evaluates residual impairment in gait stability and obstacle crossing twelve months after discharge from rehabilitation.

**Discussion:**

The results of the FLASSH project will inform falls prevention practice for stroke survivors. If the falls prevention program is shown to be effective, low cost strategies to prevent falls can be implemented for those at risk around the time of discharge from rehabilitation, thus improving safety and quality of life for stroke survivors. The two sub-studies will contribute to the overall understanding and management of falls risk in stroke survivors.

**Trial registration:**

This trial is registered with the Australian and New Zealand Clinical Trials Registry (ACTRN012607000398404).

## Background

Each year, approximately forty-eight thousand Australians are affected by stroke [1]. Despite recent advances in the management of acute stroke, including a decrease in mortality rates, stroke remains one of the leading causes of disability [1], with up to 280,000 stroke survivors in Australia experiencing disability, of whom 146,000 have a disability directly resulting from the stroke [2]. Minimising stroke sequelae is therefore important for individuals and the community.

Falls are a common adverse event among stroke survivors. It is estimated that between 14–65% fall at least once during hospitalisation [3-5] and up to 73% fall during the first six months after discharge home from hospital [6,7]. This fall rate is substantially higher than in community-dwelling older people, with approximately 30% falling in a twelve-month period [8,9]. Frequent falls appear to be more common in stroke survivors who have decreased balance or who have had a fall during their hospital admission [6].

Falls in the stroke population can have serious consequences. Estimates of soft tissue injury rate following a fall range from 24% to 48% [7,10,11]. More severe injury, such as fracture, has been reported to occur in 1% to 15% of falls in stroke survivors [7,12]. The rate of fractured neck of femur after stroke is up to two to three times higher than in the general population [13], and is in part due to the potential for decreased bone mineral density particularly in those who are unable to walk at 2 months post-stroke [14].

At present there are no published randomised controlled trials investigating the effectiveness of falls prevention strategies in stroke survivors. There is, however, evidence for the effectiveness of single and multi-factorial interventions in older community-dwelling people [15]. Exercise, including components of strength and balance, but not necessarily including a walking program, appears to be effective in preventing falls in older adults [16,17], and other interventions such as home modification and reduction in psychotropic medication have also been shown to be effective [18,19]. While it is likely that some of the approaches shown to be effective in reducing falls in older people generally may also be effective in people with stroke, there are additional stroke-specific risk factors, for example inattention or neglect, which might influence uptake and effectiveness of interventions in the stroke population. The purpose of this randomised controlled trial is to determine whether an individualised, multi-factorial intervention is effective in reducing falls in people with stroke following discharge from rehabilitation.

Fear of falling is a key factor in relation to falls [20]. Even in the absence of physical injury, a fall can have negative functional and psychological consequences, including loss of confidence or fear of falling, which can lead to activity restriction. Restricting activity may lead to further loss of strength or heightened balance impairment, which then predisposes an individual to further falls.

It has been shown that fear of falling is common in older adults [21] and although there are few published studies about the prevalence in the stroke population, it appears to be present in stroke survivors [22,23]. There is evidence to support an association between falls, fear of falling and function in older people [24] and in stroke survivors [25]. However, it is not clear whether there is a causal relationship, that is, whether a fall causes fear of falling, whether fear of falling precipitates a fall, or whether a third factor causes both. While there are several cross-sectional studies which highlight the association between falls, fear of falling and function [22,25], longitudinal studies are required to evaluate changes in fear of falling and function over time, particularly in relation to the short-term effects of a fall.

If a reduction in falls is observed, it is important to understand the mechanisms by which this has occurred. The reduction in falls may be secondary to an improvement in stability, or it may reflect increased caution when performing activities such as walking. The impact of the intervention on stability during walking is particularly pertinent, as the control of balance while walking is complex due to the inherent instability and dynamic nature of the task [26]. Examination of stability during walking will provide insight into the mechanisms by which the intervention may have an effect on reducing falls. Obstacle crossing has been shown to be a useful paradigm for exploring stability in walking following stroke [27,28].

The aims of this study are therefore threefold:

(i) to evaluate the effectiveness of a multi-factorial falls prevention program for stroke survivors returning home after rehabilitation,

(ii) to examine the temporal relationship between fear of falling, falls and function in the above population, and

(iii) to evaluate the contribution of gait impairments to the task of obstacle crossing in the context of a falls prevention program.

## Methods and design

This study consists of one main and two sub-studies. The main study is a randomised controlled trial evaluating the effectiveness of a multi-factorial falls prevention program in stroke survivors discharged home after rehabilitation. The first sub-study is a 12 month longitudinal study investigating the temporal association between falls, fear of falling and activity level, and the second sub-study is a cross-sectional study, evaluating changes in gait characteristics following stroke and their contribution to the ability to negotiate obstacles while walking.

### Randomised controlled trial

#### Participants

Stroke survivors aged forty-five years and over who have completed rehabilitation and have been discharged home will be eligible for the study if they have decreased balance or if they have fallen at any time during their hospital admission. Decreased balance is defined as a step test [29] worst leg score of less than 7 or a Berg Balance Scale [30] score of less than 49. These inclusion criteria were selected as it has been shown that decreased balance as defined above or a fall in hospital are predictors of multiple falls in the first 6 months after discharge from stroke rehabilitation [6]. Stroke sub-types will include infarct and haemorrhage (including sub-arachnoid haemorrhage) but will exclude sub-dural haemorrhage or infarct or haemorrhage due to malignancy. Those being discharged to residential care facilities and those being discharged more than one hundred kilometres from study sites will not be eligible.

#### Settings/locations

Participants will be recruited from the rehabilitation units of five health services in Melbourne and four health services in Adelaide, Australia. Participants will be assessed for eligibility by treating physiotherapy staff while still an in-patient, and if agreeable, participants will be contacted by the research team within one to two weeks of discharge. All study assessment and intervention activities will be conducted in the participant's home. Ethics approval for the study has been granted by the Human Research Ethics Committees of Melbourne Health (HREC 2006.026), Austin Health (H2006/02473), St Vincent's Health (HREC-A 066/07), Northern Health (E03006) University of South Australia (P043/06), Repatriation General Hospital (11/06), Royal Adelaide Hospital (060307), and The Queen Elizabeth Hospital (2008053). These ethics approvals cover all recruitment sites.

#### Procedures

**Figure 1:**
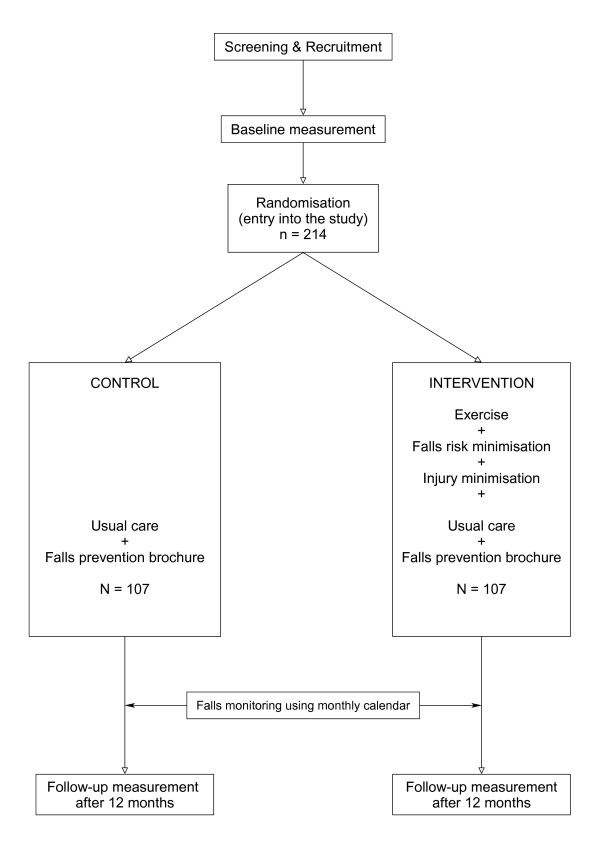
**Study design – randomised controlled trial**.

Figure 1 depicts the study design. Participants will be assessed at baseline (following recruitment) and after 12 months in the study. Baseline assessment will include the following general measures:

(i) falls risk, using the Falls Risk for Older People in the Community (FROP-Com) [31],

(ii) functional lower limb strength measured by the five chair sit-to-stand test (5-STS) [32],

(iii) gait and balance, measured by gait velocity over 5 metres at self-selected comfortable and fast pace (5mwt-comf, 5mwt-fast) [33], Step Test (ST) [29], and Timed Up and Go with and without a secondary cognitive task (TUG-single, TUG-dual) [34,35],

(iv) activity measured by the Human Activity Profile Adjusted Activity Score (HAP-AAS) [36], and

(v) function, as measured by FIM ^® ^[37].

In addition, the following stroke specific measures will be included at baseline:

(i) participation, measured by the London Handicap Scale (LHS) [38],

(ii) fear of falling, measured by the Falls Efficacy Scale (Swedish Modification (FES-S) [39,40],

(iii) inattention, assessed by the Baking Tray Task (BTT) [41] and the Star Cancellation Test (SCT) [42],

(iv) visual field, assessed by the Visual Field Confrontation Test (VFT) [43], and

(v) frontal function as measured by the Frontal Assessment Battery (FAB) [44].

#### Randomisation

Participants will be assessed for eligibility, provided with information about the study, provide informed consent, be enrolled into the study and complete the baseline assessment prior to allocation into the control or intervention group. Participants will be assigned to the control or intervention arm of the study according to a computer generated random allocation sequence concealed from the researchers in opaque envelopes. Staff not involved in the study will undertake the sequence and concealment. The envelopes containing the allocation will be stored in one location and participants will be assigned in order of completed baseline assessment.

#### Intervention

The intervention consists of a multi-factorial falls prevention program implemented by a physiotherapist including:

1) an individualised home exercise program with strength, balance and walking components based on the Otago Exercise Programme [45,46]. Exercise selection and dosage will be determined by the research physiotherapist and administered in the participant's home at a visit following the baseline assessment. Exercises will be selected to address balance and mobility problems identified in the baseline assessment and will be tailored to the level of each participant. The program will be monitored and modified by the physiotherapist at two subsequent home visits during the 12 month study period. Participants will be advised to undertake the exercise program at least five times per week. Participants will be provided with an exercise recording sheet to indicate completed exercises, and adherence to the exercise program will be assessed by the physiotherapist at the review visits.

2) falls risk minimisation strategies based on the falls risk factors identified in the baseline assessment in the context of falls prevention activities already in place, together with participant preference. Falls risk factors will be identified using the FROP-Com, which is designed to identify the level of falls risk associated with the common falls risk factors in community-dwelling older persons. Interventions may include, for example, referral for vision assessment and correction, changes to footwear, change in walking aid, referral for home modifications, medication review, and continence review and management. Guidelines for this component of the intervention are summarised in Table 1.

**Table 1:** Guidelines for intervention options for FLASSH study: falls risk minimisation

**Risk factor**	**Intervention options if risk factor identified**
History of falls/falls injuries	Referral to GP Appropriate acute management if injury recent
Medications (number)	Refer to GP for medication review
Medications (associated with increased falls risk)	Refer to GP/specialist for medication review
Medical conditions (associated with increased falls risk)	Refer to GP/specialist for review
Sensory loss: vision	Brochure on lighting and home safety Refer to optometrist Refer to Ophthalmologist via GP Refer to Vision Australia (includes mobility training and aids for visually impaired) Refer to OT for home assessment Refer to GP
Sensory loss: hearing	Refer to audiologist
Sensory loss: somatosensory	Refer to podiatrist Refer to GP
Feet and footwear	Refer to podiatrist Provide information about good footwear and falls prevention
Cognitive status	Refer to GP Memory Clinic referral in consultation with GP if memory problems have not been investigated Referral to OT for strategies to facilitate memory and/or home assessment
Continence	Refer to GP Continence clinic referral Refer to physiotherapist Refer for OT home assessment/functional assessment, including need for commode
Nutritional status	Provide brochure on nutrition Refer to dietician GP referral
Environment	Provide with information pamphlet on safety in the home Refer for OT home assessment/functional assessment and training
Function	Refer to OT for home assessment/functional assessment and training Refer to Physiotherapist for assessment and exercise to improve function Referral for support services
Functional behaviour	Refer to physiotherapist, OT, GP
Balance	Home exercise program Referral to Physiotherapist Referral for OT for a home assessment Referral to GP for further investigation
Gait and physical activity	Home exercise program Referral to Physiotherapist Referral for OT home assessment/functional assessment and training Brochure on tips for walking
Osteoporosis risk	Refer to GP for consideration of calcium and/or vitamin D supplements Refer to dietician Hip protectors
Additional options for preventing falls injury	Hip protectors Personal alarm

GP: General practitioner

OT: Occupational therapist

3) education of the patient and their carer about identified falls risk factors and risk minimisation, including provision of information in verbal and written forms. This will be undertaken by the physiotherapist implementing the exercise program.

4) injury risk minimisation strategies for those identified as having a high risk for fracture based on falls risk, delayed walking post-stroke or previous diagnosis of osteoporosis. These strategies will include prescription of hip protectors and liaison with the participant's general practitioner for consideration of vitamin D and calcium supplementation.

Participants in the intervention group will continue to receive usual care which consists of any activities undertaken by the participants recommended or administered by their treating team. In addition, intervention participants will receive a general information booklet on falls prevention (developed by Peninsula Health Falls Prevention Service, Victoria, Australia).

#### Control group

The control group will receive usual care which consists of any activities undertaken by the participants that are recommended or administered by their treating team. In addition, control participants will receive a general information booklet on falls prevention (developed by Peninsula Health Falls Prevention Service, Victoria, Australia).

#### Outcomes

The primary outcome for this study will be the incidence of falls in the 12 month period post-discharge. Based on the definition by Lamb et al [47] a fall is defined as a sudden, unexpected event in which an individual comes to rest on the ground, the floor, or other surface. A lay perspective of this definition will be provided to participants to assist with ascertainment of falls [47]. Falls will be monitored prospectively using a falls calendar supplied to participants. Participants will complete a tear-off calendar page each month marking any falls that have occurred. In addition, information about each fall will be collected such as location, activity prior to fall, injuries sustained, and medical treatment required. The calendar page will be returned to study personnel using pre-paid envelopes after the end of each month. Follow-up calls to those who do not return calendar pages within 2 weeks of the end of the month will be undertaken to ensure complete data. The researcher collating information about falls and undertaking the follow-up phone calls will be blind to group allocation.

Secondary outcomes will include the following:

(i) functional lower limb muscle strength (5-STS) [32],

(ii) gait and balance measures (5mwt-comf, 5mwt-fast [33], ST [29], TUG-single [34], TUG-dual [35])

(iii) activity level (HAP-AAS) [36],

(iv) participation (LHS) [38],

(v) function (FIM^®^) [37],

(vi) fear of falling (FES-S) [39,40],

(vii) falls risk (FROP-Com) [31].

In addition, time taken to first fall, and fall injury severity will be evaluated.

The research physiotherapist conducting the 12 month follow-up assessment will be blind to group allocation and participants will be specifically instructed not to reveal their group allocation to the researcher.

#### Sample Size

It is estimated that in the usual care group, 75% of participants will experience a fall in the 12 month follow up period (given their increased risk of falling as outlined in the project inclusion criteria [9]), and that the intervention would reduce this incidence by one third to 50%. Based on 90% power to detect a significant difference (p = 0.05, two-sided), 85 participants are required for each arm of the study. Allowing for a 25% dropout rate, we plan to enrol 107 participants in each group giving a total sample of 214.

#### Statistical methods

Intention to treat analysis will be used. To compare falls in the intervention and control groups, negative binomial regression will be used. This model is appropriate as falls are recurrent events which are not normally distributed and follow up time is different for individuals [48]. Fall data will also be summarised as the fall rate per person year, time to first fall, as well as the number of falls, number of fallers, non-fallers and frequent fallers (two or more falls) [47]. Repeated measures analysis of variance will be used to determine differences in secondary outcomes between control and intervention groups from baseline to follow up, with a p-value set at < 0.05.

### Fear of Falling Sub-Study

#### Procedures

In this longitudinal study, FLASSH participants from both the intervention and the control groups recruited from the sites in Melbourne, Australia will be included. This is estimated to be approximately half the overall project sample. Participants will be assessed at baseline, at four monthly intervals, and following any fall throughout the 12 month study period by a researcher blind to group allocation. Assessments will be conducted in the participant's home, and at each time-point, the following will be assessed:

(i) fear of falling (FES-S) [39,40]

(ii) gait and balance (5mwt [33], TUG (single and dual tasks) [34,35], Step Test [29])

(iii) activity (HAP-AAS [36]).

#### The study outcomes

The primary outcomes for this study will be fear of falling (FES-S), gait (gait velocity, TUG-single and dual), balance (ST), and activity level (HAP-AAS).

#### Sample size

It is estimated that approximately half of the overall sample will be included in this sub-study, giving a sample size of 80. The sample will include participants from the control and intervention arms of the main study.

#### Statistical methods

Descriptive statistics and multivariate analysis of variance will be used to determine changes in fear of falling and physical function between control and intervention groups, and faller and non-faller groups over time. Time series analyses and individual case analysis will be used to identify links between fall events and changes in fear of falling.

### Obstacle Crossing Sub-Study

Participants recruited in the primary study from both intervention and control groups through Melbourne sites will be approached to participate in an additional measurement session at twelve months following enrolment in the study. It is anticipated that 30 subjects will be recruited for this sub-study.

#### Apparatus

A VICON^® ^motion analysis system and AMTI forceplates will be used to collect kinematic and kinetic data. Data will be processed and analysed using BodyBuilder^® ^software, which is designed for use with the VICON^® ^system.

#### Procedure

During testing, subjects will wear shorts and singlet to allow marker application. They will also wear well-fitting shoes and any prescribed eyewear usually worn during ambulation. Reflective markers will be placed on the lower limbs and trunk using double sided adhesive tape. Motion of the reflective markers will be recorded by the VICON^® ^motion analysis system. Data on centre of pressure (COP) will be collected using the AMTI forceplates. Subjects will perform three or four walks at a comfortable speed. They will then be asked to perform eight trials where they walk and step over a 4 cm high obstacle, placed in the middle of the walkway. Subjects will be cautioned to perform the tasks within their limits of safety and stop if they feel at risk at any time.

#### Outcome Measures

Based on results of previous studies [27,28], the primary outcome measures will be

(i) lead limb post obstacle distance,

(ii) lead and trail limb clearance,

(iii) step length after clearing the obstacle,

(iv) anterior-posterior (AP) separation between the centre of mass (COM), COP and heel of the stance limb during lead limb clearance, and

(v) instantaneous COM velocity at lead limb clearance.

#### Statistical Analysis

Data will be compared with normative data using multivariate analysis of variance. Adjustment will be made for any covariates which may impact on obstacle crossing, such as leg length. Performance on the primary gait measures will be compared between participants in the experimental and control groups of the randomised controlled trial, using multivariate analysis of variance, with adjustment for covariates identified as differing between the two groups at this measurement occasion.

## Discussion

Despite evidence of the effectiveness of falls prevention activities for community-dwelling older people, the applicability of these interventions has not, to date, been evaluated in community-dwelling stroke survivors. Interventions related to falls risk factors, for example, strength and balance training, have been shown to be effective in the stroke population in achieving improved physical performance, but the impact on falls has not always been reported. This study aims to address this gap as well as providing further insights into how fall-related self-efficacy changes over time, and the contribution of gait impairments to stability when negotiating obstacles. If the study shows that the intervention is effective in reducing falls, the results will provide a sound basis for broad implementation of a falls prevention program that could be commenced prior to discharge from hospital with relatively little additional resources required. Successful outcomes from the studies have the potential to result in improved safety and independence among people returning home after stroke.

## Competing interests

The authors declare that they have no competing interests.

## Authors' contributions

KDH, SFM, CMS and CW conceived the idea, developed the overall study design, and obtained funding for the study. FAB was involved in refining the design of the study and drafted the manuscript. All authors critically reviewed the manuscript and approved the final manuscript.

## Pre-publication history

The pre-publication history for this paper can be accessed here:


